# Genetic Population Structure Analysis in New Hampshire Reveals Eastern European Ancestry

**DOI:** 10.1371/journal.pone.0006928

**Published:** 2009-09-07

**Authors:** Chantel D. Sloan, Angeline D. Andrew, Eric J. Duell, Scott M. Williams, Margaret R. Karagas, Jason H. Moore

**Affiliations:** 1 Computational Genetics Laboratory, Department of Genetics, Dartmouth Medical School, Lebanon, New Hampshire, United States of America; 2 Community and Family Medicine, Dartmouth Medical School, Lebanon, New Hampshire, United States of America; 3 Norris Cotton Cancer Center, Dartmouth Medical School, Lebanon, New Hampshire, United States of America; 4 Lifestyle, Environment and Cancer Group, Genetics and Epidemiology Cluster, International Agency for Research on Cancer, Lyon, France; 5 Unit of Nutrition, Environment and Cancer, Cancer Epidemiology Research Programme Institut Català d'Oncologia (ICO) Catalan Institute of Oncology, L'Hospitalet de Llobregat, Barcelona, Spain; 6 Center for Human Genetics Research, Vanderbilt University, Nashville, Tennessee, United States of America; University of California San Diego, United States of America

## Abstract

Genetic structure due to ancestry has been well documented among many divergent human populations. However, the ability to associate ancestry with genetic substructure without using supervised clustering has not been explored in more presumably homogeneous and admixed US populations. The goal of this study was to determine if genetic structure could be detected in a United States population from a single state where the individuals have mixed European ancestry. Using Bayesian clustering with a set of 960 single nucleotide polymorphisms (SNPs) we found evidence of population stratification in 864 individuals from New Hampshire that can be used to differentiate the population into six distinct genetic subgroups. We then correlated self-reported ancestry of the individuals with the Bayesian clustering results. Finnish and Russian/Polish/Lithuanian ancestries were most notably found to be associated with genetic substructure. The ancestral results were further explained and substantiated using New Hampshire census data from 1870 to 1930 when the largest waves of European immigrants came to the area. We also discerned distinct patterns of linkage disequilibrium (LD) between the genetic groups in the growth hormone receptor gene (GHR). To our knowledge, this is the first time such an investigation has uncovered a strong link between genetic structure and ancestry in what would otherwise be considered a homogenous US population.

## Introduction

Genetic population structure is the presence of genetically distinct subgroups that result from shared ancestry within a larger population. Most notably, structure was displayed by Rosenberg et al., when the Bayesian clustering method *structure* was used to group 1056 individuals from 52 populations, using microsatellite data [1]. This “large-scale” genetic structure was further corroborated by Li et al. in 2008, in an analysis of 650,000 SNPs from the Human Genome Diversity panel [2]. Other researchers have continued to investigate regional structure patterns with a variety of results [3]–[14]. Of particular interest is that even in presumably homogeneous populations, genetic structure has been detected and linked to geography [15], [16]. These studies of genetic structure are important because they can be used to prevent confounding in genetic epidemiology studies and are key to elucidating the genetic anthropology of a region.

There have been several studies exploring the link between genetic structure and shared ancestry [1], [17]–[19]. Most of these studies within evolutionary population genetics (unlike those used to ascertain confounding in genetic epidemiology) focused on the structure of ethnic groups with clearly distinct histories or geographical locations (i.e. Caucasian, African-American, Hispanic, Asian), and did not find additional reliable subdivision. They also typically begin with the ascertainment of each individual's ancestral population history and then use those population groups to supervise the clustering methods. These studies provide tremendous insight into population genetics and human evolution. However, as previously mentioned, subgroups have been identified within presumably homogeneous or highly admixed populations, suggesting that a subset of individuals share some ancestry. The question therefore becomes whether individuals identified within a genetic subgroup can later also be associated with a particular geographic ancestry. Subsequently, do these genetic and ancestral subgroups provide more information about a region's history than currently available methods such as census records? If ancestral and genetic subgroups can be ascertained, it is also important for genetic association studies taking place in that region because typical self-reported race data may not adequately control for substructure confounding.

The state of New Hampshire is an ideal place to investigate these questions because it is highly admixed, with what is generally considered predominantly Western European and French-Canadian inhabitants. However, the state is usually considered ancestrally homogeneous from the viewpoint of epidemiological studies, with 96% of citizens being Caucasian (2000 census, http://www.census.gov/main/www/cen2000.html). There is also a wealth of historical and census data that can lend insight into predominant immigration patterns.

## Results

**Figure 1:**
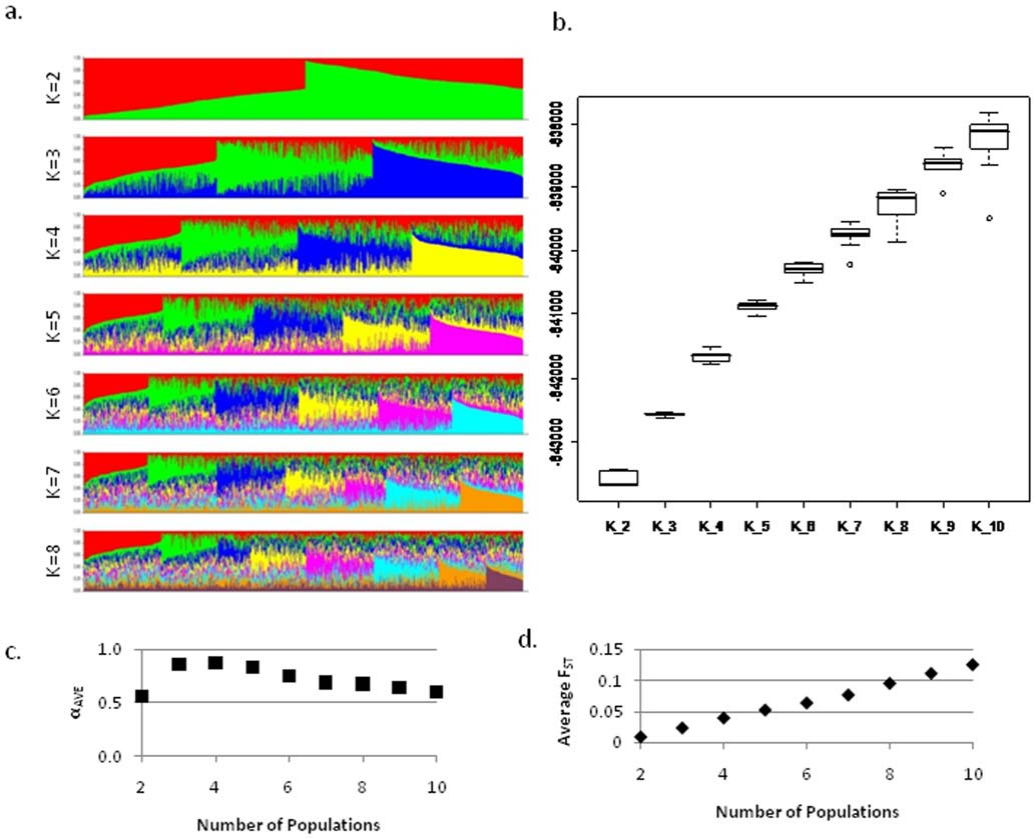
Bayesian clustering results. a) Bar plots from *CLUMPP* results aligning 10 *structure* runs for K = 2 to K = 10. Each plot was created using 960 tagSNPs from 864 individuals, and is sorted by q values. The plots are read from left to right, with bars representing individuals and the color of the bar representing the proportion of that individuals' markers that originated from a certain population. b) Probabilities from *structure* shown as boxplots of the 10 runs at each K. *Structure* admixture (c) and F_ST_ (d) values for 10 runs. The F_ST_'s graphed are averages across each subpopulation for each K.

This study is based on controls enrolled in the New Hampshire Bladder Cancer and Skin Cancer Studies (n = 864) conducted at Dartmouth Medical School [20]. Subjects were genotyped for 1529 single nucleotide polymorphisms (SNPs) within suspected cancer susceptibility genes, though filtering for SNPs that would unduly influence the clustering results (those in linkage disequilibrium at r^2^ of 0.8) reduced the number of SNPs to 960 within 360 genes. There were between 1 and 13 SNPs per gene with an average of 2.7 and median of 2 (Table S1). The genotype data are more fully described in [20], [21]. Bayesian clustering conducted using the *structure* software revealed distinct subpopulations, with the highest and most reliable probabilities between a K of 5 and 7. The bar plots are shown for K = 2 to K = 8 from the *CLUMPP* software (aligns multiple runs of *structure*) from 10 runs at each K (Figure 1a). As expected, individuals in the sample appear highly admixed; however distinct populations are discernible. The FST's increase consistently as K increases, with the average FST's for K = 4 to K = 7 around the level of “little genetic differentiation” as defined by Wright (approx. 0.05) (Figure 1c,d) [22]. The admixture values increase for lower K's, but begin to drop at K = 6 to values between 0.6–0.7 (Table S2). In selecting the most correct K, parsimony is an important consideration, i.e. that the simpler answer tends to be correct. Though there may be some validity to further subdividing the groups, the most statistically consistent and the most parsimonious K based on the *structure* output is K = 6. Further analysis using the ancestral data is used to describe the groupings and lends support to our selection of K = 6.

The overall results from a Spearman's rank correlation between self-reported ancestry for each individual and their structure q values (the proportion of their SNPs from each population) are given as p-values in Tables 1 and S3 (Table S3 is full results, Table 1 shows only significant results) for K = 3 to K = 7. Of particular interest is the consistency with which Lithuania, Poland and Russian ancestries correlate, forming a distinct and single group, as well as the strong ancestry of Finland, which represents a clear group for K = 4 through K = 7. Sample sizes as well as an investigation of individual reporting of ancestries and which population each person is assigned to based on their maximum q values for K = 6 is shown in Table S4. Lithuania, Russia and Finland all have fairly small sample sizes (n = 12, 13, 7), though Poland's sample size is larger with 44 people reporting Polish ancestry. Of these, 7 people reporting full Polish ancestry and 9 part Polish ancestry have their maximum q values for *structure* runs for population 6. Of the 7 people reporting Finnish ancestry, 4 have their maximum q values for population 5, with their average q's being relatively high (0.52). The Czech population is the smallest that significantly correlates with a population group; 2 of the 5 individuals assigned to population 3. The groups for which there were larger sample sizes less clearly correlate with different structure groups, such as England with population 2 and France with population 4, though these also have mixed historical ancestries. This is somewhat expected as these larger groups make up those that helped to originally settle New Hampshire, and therefore form the genetic background with which the other, smaller ancestral groups admixed. The Canadian Indians, French and Jewish population groupings seem similarly complex. However, it has been noted in a previous study that a New York City Jewish population tended to group with Southern Europeans, demonstrating a strong Mediterranean influence [23]. There may also be French Canadian mixing with the Canadian Indian group, so that in essence those of both the Jewish and Canadian Indian ancestry share some Southern European influence. However, this will require further investigation.

**Table 1:** Ancestry analysis results for between 2 and 7 populations assumed.

Number of Populations	Population Group	Ancestries (p-value)		
K3	1	Finland (0.005)	Ireland (0.05)	
	2	Italy (0.022)	UK (0.027)	
	3	Ca_Indian (0.005)	Germany (0.026)	Russia (0.019)
K4	1	Poland (0.015)	Russia (0.001)	UK (0.035)
	2	Ca_Indian (0.008)	Jewish (0.049)	
	3	Finland (0.008)	Switzerland (0.038)	
	4	Italy (0.017)	Netherlands (0.024)	
K5	1	England (0.011)	Italy (0.01)	Netherlands (0.004)
	2	Lithuania (0.037)	Poland (0.001)	Russia (0.000)
	3	Ca_Indian (0.016)	France (0.035)	Jewish (0.037)
	4	Am_Indian (0.04)	Ca_Indian (0.035)	Canada (0.025)
	5	Finland (0.006)	Switzerland (0.043)	
K6	1	Am_Indian (0.043)		
	2	England (0.024)	Italy (0.03)	Netherlands (0.002)
	3	Czech (0.029)		
	4	Ca_Indian (0.021)	France (0.02)	Jewish (0.023)
	5	Finland (0.006)		
	6	Lithuania (0.017)	Poland (0.001)	Russia (0.001)
K7	1	Ca_Indian (0.011)		
	2	England (0.03)	Italy (0.005)	Netherlands (0.007)
	3	Am_Indian (0.027)	UK (0.038)	
	4	Finland (0.007)		
	5	Czech (0.029)		
	6	Lithuania (0.024)	Poland (0.001)	Russia (0.001)
	7	Ca_Indian (0.046)	France (0.012)	Jewish (0.023)

A Spearman's rank correlation between each ancestry with more than 5 individuals reporting For each population, ancestries with a Spearman's rank correlation p-value<0.05 are shown along with their p-values (in parenthesis).

The finding that Eastern European ancestries correlate with distinct genetic subpopulations in New Hampshire was surprising. Finland has a unique genetic history with a known strong founder effect and also showed a strong signal in the previously mentioned New York City study. Sweden is the most well-known historic contributor to Finland genetics, however it is Switzerland that clusters with Finland at K = 4 and K = 5 in our investigation [24]. The ancestry results lend support to a model of K = 6, as the divisions between, e.g. Finland and the rest of the population are more clear than lower K's, and K = 7 is less clear as Canadian Indian ancestry appears in two separate populations (complete Table S3), (although this may represent subdivisions within the Canadian Indian group).

**Figure 2:**
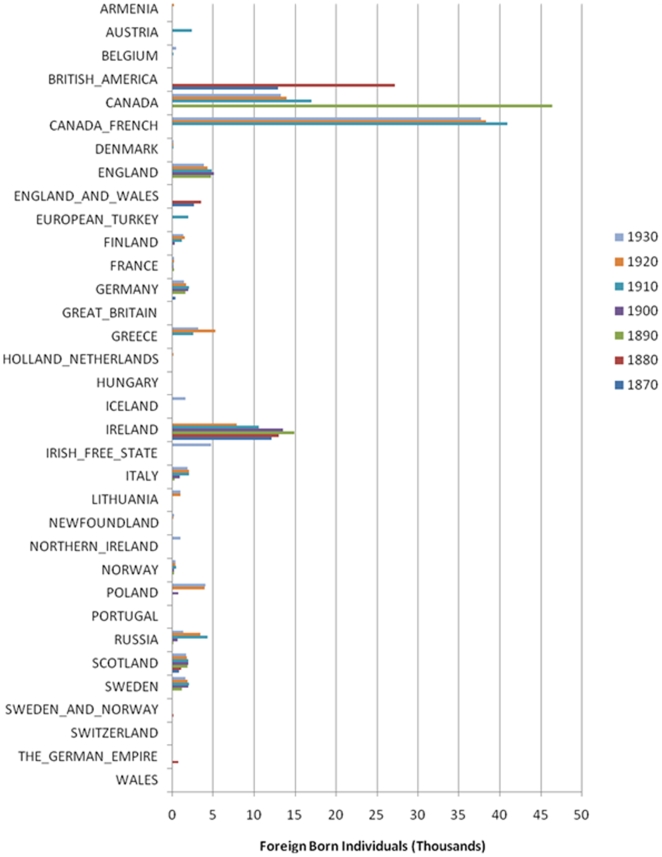
Census data for New Hampshire from 1870 to 1930 showing thousands of immigrants from European countries by census year.

New Hampshire census data from 1870 to 1930 is the most effective time period to investigate, because around the turn of the 20th century there was a great deal of immigration to New Hampshire from all over Europe, Canada and elsewhere in New England [25] (Figure 2). The immigrants predominantly moved into the mill towns such as Manchester and Milford located in the south-central region (Hillsborough County) to find employment. The census demonstrates that the largest single group came from Canada, many of whom were French Canadian. Major immigrant groups also came from Ireland, England and Scotland. These populations also constituted the bulk of earlier immigration. Smaller, though not inconsequential immigrant groups arrived from Germany, Russia, Greece, Sweden, Poland, Lithuania and Finland along with other European countries. In 1930 there were 1427, 4101, 1084 and 1386 individuals in New Hampshire who were respectively born in Russia, Poland, Lithuania and Finland. Our data demonstrate that these groups influenced the state's genetic substructure. The Czech group is interesting, despite the small sample size, because Czechoslovakia was not founded until 1918. Therefore, data on individuals born in Czechoslovakia were not recorded in the United States census during most of the large waves of immigration. The self-reported ancestral and genetic structure data lend evidence to a genetic contribution of the region of the current Czech Republic to New Hampshire despite the lack of historical record, though more study on this topic is required due to the very small sample size of the Czech group. Further investigation of the census data shows that most of the immigrants were moving to Hillsborough County likely in pursuit of jobs at the mills that were being built there during the industrial revolution (Figure S1). A few groups seemed to selectively migrate to other regions of the state, such as the Norwegians largely settling in Coos County (in the north). Geographical analysis supports the intuition that the most genetically diverse places are in high population areas (Figure S2).

**Figure 3:**
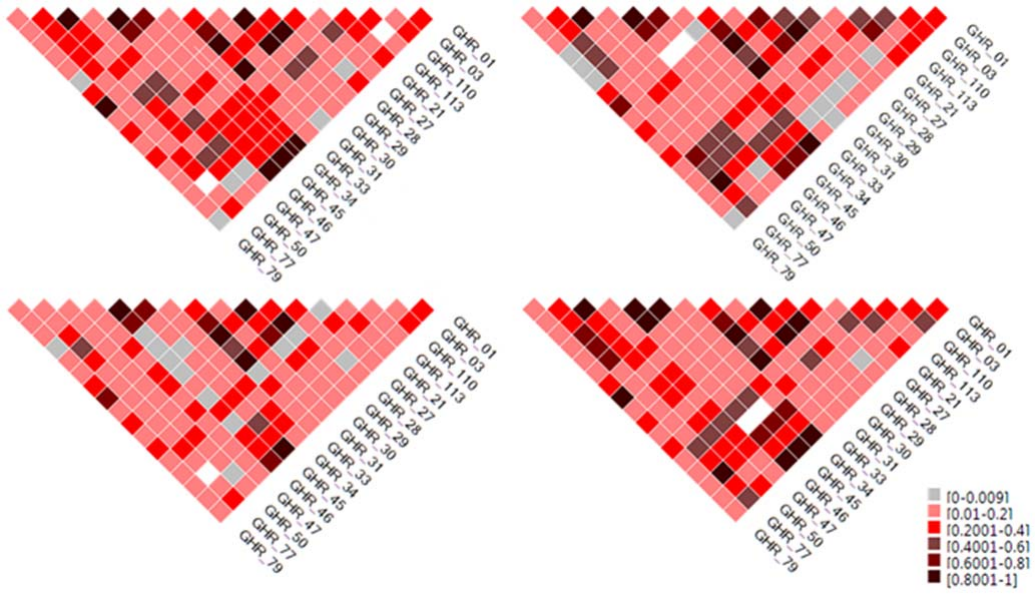
D' values using 18 SNPs from the GHR gene for K = 4 population clusters.

Plots of D′ revealed different LD patterns among the genetic population subgroups in the growth hormone receptor (GHR) gene at K = 4 (Figure 3). For K = 4 the LD plots shows visual differences especially between population 3 and the other populations. This population corresponds to the Finland/Switzerland ancestry group. Plots above K = 4 are difficult to compare due to missing data, as we restricted the analysis to those individuals that could be absolutely placed in one population (q> = 0.5001). Statistical haplotype comparisons determined that there is statistically significant association between haplotypes and population membership between populations one and two, corresponding with the Poland/Russia/UK group and the Canadian Indian/Jewish group (Table 2). Other comparisons were not significant when corrected for multiple testing.

**Table 2:** Haplotype association analysis results using score statistics as computed within the R package haplo.stats.

	*global*	1	2	3	4
1 (n = 49)	*0.01542*	NA	0.0001*	0.05783	0.00626
2 (n = 89)	*0.00139*		NA	0.00067	0.24951
3 (n = 80)	*0.0081*			NA	0.00804
4 (n = 60)	*0.35765*				NA

Global values were obtained by comparing individuals from each population to all others from the other 3 populations. Subsequent p-values presented are obtained by comparing haplotypes between groups. The haplotypes were associated when comparing populations 1 and 2*, with a p-value below a Bonferroni corrected alpha of 0.000347.

## Discussion

These results suggest that genetic population structure is detectable in a highly admixed US population and that this structure correlates with self-reported ancestry. To our knowledge, this is the first time such an investigation has uncovered a strong link between structure and ancestry in what would otherwise be assumed to be a homogeneous US state where most individuals are of European ancestry. Our data indicate that that admixture has not eliminated the genetic structure found within Europe, and descendants of the Russian, Polish and Lithuanian immigrants remain genetically distinct from the rest of the population and are closely related to one another. These results are unique in that they are analyzed on an individual, rather than population basis, and use a relatively small number of SNPs compared to Genome-wide studies. Of further interest is the fact that these findings are based on a panel of SNPs in hypothesized cancer susceptibility genes. Since the clustering was done within cancer susceptibility genes, subsequent investigation may reveal a different general cancer susceptibility subtype (and thus disease risk) in each of these genetic and ancestral sub-populations. Such patterns of variation indicate that investigators undertaking genetic epidemiology research in New Hampshire, the larger New England region or other areas of the United States where there is a known Eastern European influence should consider taking self-reported ancestry into account to avoid structure influencing their results.

## Materials and Methods

### Data collection

Controls less than 65 years of age were selected using population lists obtained from the New Hampshire Department of Transportation. Controls 65 year of age and older were chosen from data files provided by the Centers for Medicare & Medicaid Services (CMS) of New Hampshire. We interviewed a total 1191 controls throughout the state, of which 70% were confirmed to be eligible for the study. Informed consent was obtained from each participant and all procedures and study materials were approved by the Committee for the Protection of Human Subjects at Dartmouth College. Consenting participants underwent a detailed in-person interview, usually at their home. Subjects were asked to provide a blood sample (buccal sample was requested if a blood sample could not be drawn).

Genotyping was performed on all DNA samples of sufficient concentration (864 control individuals) using the Golden Gate Assay system by Illumina's Custom Genetic Analysis service (Illumina, Inc., San Diego, CA). Samples repeated on multiple plates yielded the same call for 99.9% of SNPs and 99.5% of samples submitted were successfully genotyped. Genotype calls were 99% concordant between genotyping platforms (Taqman). We obtained genotype information from 1529 single nucleotide polymorphisms (SNPs) in suspected cancer susceptibility genes scattered throughout the genome. After filtering the data for SNPs in Hardy-Weinberg disequilibrium, we used the tagSNP software within Haploview to tagSNP the data (r^2^ = 0.8) to be sure that the clustering was not driven by LD. The 960 remaining tag SNPs were then used in the *structure* analysis. Only control individuals (no history of bladder cancer) were used in this study to prevent case/control status from confounding the analysis.

### Bayesian Clustering

In order to determine if genetic subpopulations are present in the New Hampshire population we used Bayesian clustering as implemented in the *structure* program to cluster individuals using the remaining 960 SNPs. *Structure* iteratively clusters based on a user-supplied “K” number of populations. The genotype data were analyzed using the *structure*(v. 2.2.3) admixture model, without population data assigned (burnin of length 30,000, followed by 100,000 iterations) for 10 repetitions of each K from 2 to 10 [26]–[28]. This is far beyond the default number of iterations for *structure*, but high consistency between runs even at large K's were observed at values higher than the default. We concurrently ran random genotype data as well as the sample data from the *structure* software website as positive and negative controls. *CLUMPP* (v. 1.1.1) was used to align the repetitions for each K, using G′. The output from *CLUMPP* was used for both the ancestry and LD analyses.

### Ancestry Analysis

Once the Bayesian clustering was complete, self-reported ancestry was assessed for association with the genetic subgroups. Each study individual was asked to report the previous 2 generations of ancestral information (i.e., parents and all grandparents). Each individual in the dataset was surveyed regarding their ancestry. They were allowed to provide up to three ancestries for each of their parents and all of their grandparents. Ancestries were reported as Surveillance, Epidemiology, and End Results (SEER) country codes [29]. Exploratory analysis revealed that among the ancestries, those reported by at least five individuals were: American Indian (n = 32), Austria (n = 5), Belgium (n = 5), Canadian Indian (n = 14), Canada (n = 113), Czech Republic (n = 5), England (n = 355), Finland (n = 7), French-Canadian (n = 54), France (n = 173), Germanic (countries where Germanic languages spoken) (n = 5), Germany (n = 110), Greece (n = 9), Ireland (n = 218), Italy (n = 41), Jewish (n = 6), Lithuania (n = 12), Canadian Maritime Provinces (n = 6), Netherlands (n = 25), Poland (n = 44), Russia (n = 13), Scotland (n = 157), Sweden (n = 24), Switzerland (n = 7), UK (n = 11), US (n = 42), Wales (n = 24). The level of completeness of the data varied between the individuals; therefore we decided to undertake an individual-based analysis. Each subject's data was coded as 0,1 or 2 for each ancestry, indicating not having the ancestry at all, reporting being “part” that ancestry, or reporting only that ancestry, respectively. For instance, if a subject reported only being from England, they would be given a 2 for England and a 0 for other ancestries. Whereas a subject reporting one grandparent from England and three grandparents from France would be given a 1 for England, a 1 for France, and 0 for the others. This “none”, “part” and “all” coding could be made with more certainty than assigning weights based on the number of times an ancestry was reported. A Spearman's Rank Correlation was then calculated between the ancestry codes and the individual's q value for each population from the *CLUMPP* output for each population.

We next sought to more directly determine if individuals from the correlated ancestries historically immigrated to New Hampshire in large enough numbers to impact its current genetic makeup. Census data from 1870–1930 were obtained from the Inter-university Consortium for Political and Social Research and analyzed using the University of Virginia Historical Census Browser (http://fisher.lib.virginia.edu/collections/stats/histcensus/).

### Linkage Disequilibrium

Using a subset of the data with high LD removed, we were able to find genetic clustering using Bayesian clustering. A subsequent question was whether distinct patterns of LD could be discerned within subpopulations using the full dataset. Patterns within individual genes would lend further support or explanation to our model, as LD is known to be highly influenced by personal ancestry. The genotyped SNPs were distributed evenly throughout the genome, focusing on suspected cancer susceptibility genes. The 6 genes with the most assayed SNPs (CYP19A1, GHR, GSK3B, KRAS, PGR, PMS1, TNKS) were used to compare LD between the clusters. D' was calculated using *Powermarker* [30]. Individuals had to have a q value of at least 0.5001 in order to be included as part of a for the LD analysis. Other genes were entirely in LD for all populations or did not differentiate between populations (data not shown).

In order to statistically compare LD between each of these four populations, an association analysis between haplotypes and population membership was conducted between each of the populations and between each population and all the individuals in other populations. The analysis was conducted in R using the haplo.stats package which conducts association between traits and haplotypes using score statistics as estimated by an expectation-maximization algorithm [31].

## Supporting Information

Table S1 (0.24 MB DOC)

Table S2 (0.04 MB DOC)

Table S3 (0.10 MB DOC)

Table S4 (0.07 MB DOC)

Figure S1 The average number of European immigrants into New Hampshire from 1870 to 1930 reported as percentages of immigrants from each country moving into each county.
(0.32 MB DOC)

Figure S2 Genetic distance between individuals in New Hampshire using distance values calculated in Alleles in Space, and smoothed using kriging within ArcMap 9.3 (also shows NH county lines). Genetic distances were calculated as the number of mismatched SNPs between individuals connected in a Delaunay triangulation network divided by the total number of SNPs and assigned to the midpoint of the connecting line between individuals.
(0.34 MB DOC)
