# Supplementary material for: Genetic Population Structure Analysis in New Hampshire Reveals Eastern European Ancestry
Source: PLoS One. 2009 Sep 7;4(9):e6928. doi: 10.1371/journal.pone.0006928 (PMC2734429; doi:10.1371/journal.pone.0006928)
Supplement: Table S1 — (0.24 MB DOC) [file pone.0006928.s001.doc]

| **GENE** | **#** | **CHR** | **START** | **STOP** | **GENE** | **#** | **CHR** | **START** | **STOP** | **GENE** | **#** | **CHR** | **START** | **STOP** |
| --- | --- | --- | --- | --- | --- | --- | --- | --- | --- | --- | --- | --- | --- | --- |
| ABCA1 | 5 | 9 | 14884009 | 14942072 | ERCC1 | 1 | 19 | 18192580 | 18192580 | MYNN | 1 | 3 | 75992743 | 75992743 |
| ABCA5 | 1 | 17 | 1169561 | 1169561 | ERCC2 | 1 | 19 | 18124362 | 18124362 | MYO5A | 3 | 15 | 23434121 | 23462341 |
| ABCA6 | 2 | 17 | 1034634 | 1036166 | ERCC3 | 2 | 2 | 16741069 | 16754290 | NCF2 | 2 | 1 | 33934391 | 33943874 |
| ABCA7 | 2 | 19 | 991751 | 993524 | ERCC4 | 2 | 16 | 5342112 | 5351631 | NCOA3 | 3 | 20 | 11321401 | 11327093 |
| ABCB1 | 2 | 7 | 12448786 | 12463109 | ERCC5 | 3 | 13 | 16594193 | 16617678 | NFKB1 | 5 | 4 | 27918904 | 28030988 |
| ABCB11 | 2 | 2 | 20012011 | 20092635 | ERCC6 | 1 | 10 | 1471569 | 1471569 | NFKBIE | 3 | 6 | 35084445 | 35094103 |
| ABCC2 | 4 | 10 | 20291104 | 20352532 | ESR1 | 7 | 6 | 56280294 | 56576704 | NINJ1 | 2 | 9 | 3205070 | 3205163 |
| ABCC4 | 2 | 13 | 8905211 | 8948672 | ESR2 | 2 | 14 | 45699569 | 45768055 | NOS2A | 2 | 17 | 833467 | 833591 |
| ABCG8 | 2 | 2 | 22895539 | 22920858 | EXO1 | 2 | 1 | 6787940 | 6793171 | NOS3 | 2 | 7b | 50051985 | 50058257 |
| ADH1C | 4 | 4 | 24755493 | 24763749 | FANCA | 5 | 16 | 1366964 | 1410097 | NQO1 | 2 | 16 | 23357959 | 23366572 |
| AHR | 3 | 7b | 16704367 | 16745061 | FBXW7 | 3 | 4 | 77766095 | 77790006 | NR1H4 | 2 | 12 | 24404909 | 24437569 |
| AHRR | 2 | 5 | 427862 | 428102 | FOS | 3 | 14 | 56745379 | 56747011 | NUBP2 | 1 | 16 | 1776256 | 1776256 |
| AKR1C3 | 9 | 10 | 5044290 | 5099453 | FOXC1 | 5 | 6 | 1546773 | 1553685 | OCA2 | 3 | 15 | 633086 | 638762 |
| AKR1C4 | 1 | 10 | 5187784 | 5187784 | FUT2 | 1 | 19 | 21475447 | 21475447 | OGG1 | 1 | 3 | 9741080 | 9741080 |
| AKT1 | 1 | 14 | 86240939 | 86240939 | FZD7 | 3 | 2 | 53106465 | 53113222 | OPRD1 | 2 | 1 | 355421 | 383346 |
| ALAD | 3 | 9 | 23472395 | 23475104 | GATA3 | 6 | 10 | 2460264 | 2474517 | OPRM1 | 4 | 6 | 58515647 | 58569423 |
| ALDH2 | 1 | 12 | 2781342 | 2781342 | GC | 1 | 4 | 1125344 | 1125344 | P2RX7 | 1 | 12 | 12191748 | 12191748 |
| ALOX12 | 1 | 17 | 6500108 | 6500108 | GDF15 | 2 | 19 | 9759826 | 9759943 | PAK6 | 4 | 15 | 11323141 | 11359380 |
| ALOX15 | 1 | 17 | 4148591 | 4148591 | GGH | 2 | 8 | 15786543 | 15805034 | PARP1 | 3 | 1 | 2727118 | 2766027 |
| ALOX5 | 5 | 10 | 3271341 | 3327569 | GHR | 8 | 5 | 42378268 | 42691996 | PARP4 | 4 | 13 | 5989441 | 6055859 |
| AMACR | 7 | 5 | 33962275 | 33980857 | GPX2 | 4 | 14 | 46403869 | 46410300 | PCNA | 2 | 20 | 5039516 | 5039636 |
| APAF1 | 4 | 12 | 22525398 | 22602024 | GPX3 | 3 | 5 | 11560850 | 11570392 | PCTP | 2 | 17 | 12480530 | 12480594 |
| APC | 4 | 5 | 14577867 | 14591769 | GPX4 | 2 | 19 | 1042211 | 1046477 | PGR | 13 | 11 | 4463889 | 4586698 |
| APEX1 | 2 | 14 | 1922989 | 1924994 | GSK3B | 7 | 3 | 26090649 | 26304751 | PHB | 1 | 17 | 6134652 | 6134652 |
| APOA2 | 3 | 1 | 11682671 | 11684038 | GSTA4 | 4 | 6 | 43701012 | 43707461 | PIM1 | 2 | 6 | 27992626 | 28000212 |
| APOB | 5 | 2 | 41215 | 83144 | GSTM3 | 3 | 1 | 6365717 | 6365936 | PIN1 | 4 | 19 | 1207981 | 1221680 |
| APOE | 1 | 19 | 17677385 | 17677385 | GSTP1 | 2 | 11 | 12658484 | 12659374 | PLA2G2A | 1 | 1 | 3129304 | 3129304 |
| ARHGDIB | 2 | 12 | 7854136 | 7874075 | GSTZ1 | 1 | 14 | 58794036 | 58794036 | PLK1 | 1 | 16 | 15005484 | 15005484 |
| ARNT | 2 | 1 | 1302067 | 1340390 | HFE | 3 | 6 | 16949430 | 16954182 | PMS1 | 6 | 2 | 40858221 | 40936947 |
| ATM | 4 | 11 | 11705598 | 11797252 | HIF1AN | 1 | 10 | 21054491 | 21054491 | PMS2 | 3 | 7b | 5394028 | 5405604 |
| ATP1B2 | 2 | 17 | 7143482 | 7156811 | HMGCR | 1 | 5 | 25240613 | 25240613 | POLB | 2 | 8 | 12516830 | 12533645 |
| AXIN2 | 6 | 17 | 22252344 | 22283332 | HSD17B1 | 2 | 17 | 4429396 | 4430569 | POLD1 | 1 | 19 | 23170521 | 23170521 |
| BAK1 | 3 | 6 | 24398942 | 24405690 | HSD17B2 | 2 | 16 | 35732971 | 35743551 | POT1 | 1 | 7 | 23887321 | 23887321 |
| BARD1 | 3 | 2 | 65841608 | 65854961 | HSD17B4 | 6 | 5 | 21175428 | 21282186 | PPARG | 3 | 3 | 12333125 | 12405488 |
| BAX | 2 | 19 | 21728066 | 21733574 | HSD3B1 | 2 | 1 | 16140890 | 16144097 | PPP1R13L | 1 | 19 | 18151180 | 18151180 |
| BCL2L1 | 1 | 20 | 483420 | 483420 | HSD3B2 | 2 | 1 | 16042588 | 16051679 | PTEN | 2 | 10 | 8402202 | 8475261 |
| BCL6 | 4 | 3 | 93937685 | 93954246 | HSPB8 | 1 | 12 | 10201816 | 10201816 | PTGS1 | 1 | 9 | 32464996 | 32464996 |
| BHMT | 2 | 5 | 29010774 | 29021566 | HTR1B | 2 | 6 | 15992431 | 15993452 | PTGS2 | 4 | 1 | 37051997 | 37059690 |
| BIC | 8 | 21 | 12597384 | 12610181 | HTR1D | 3 | 1 | 6342866 | 6345682 | PTH | 3 | 11 | 12301294 | 12301746 |
| BIRC2 | 1 | 11 | 5797557 | 5797557 | HUS1 | 1 | 7 | 47389981 | 47389981 | RAB15 | 3 | 14 | 46411252 | 46412031 |
| BIRC3 | 2 | 11 | 5765267 | 5770806 | ICAM1 | 4 | 19 | 1645339 | 1659138 | RAC1 | 1 | 7 | 5798751 | 5798751 |
| BLM | 5 | 15 | 6255893 | 6318646 | IFNAR2 | 1 | 21 | 20276125 | 20276125 | RAD23B | 3 | 9 | 17402223 | 17407354 |
| BRCA1 | 3 | 17 | 4947390 | 4976227 | IFNG | 1 | 12 | 30694715 | 30694715 | RAD51 | 4 | 15 | 11776794 | 11785892 |
| BRCA2 | 5 | 13 | 13870572 | 13909232 | IFNGR1 | 2 | 6 | 41624017 | 41624209 | RAD52 | 2 | 12 | 876074 | 876940 |
| BRIP1 | 5 | 17 | 18414057 | 18593880 | IFNGR2 | 1 | 21 | 20471563 | 20471563 | RAD54L | 1 | 1 | 563292 | 563292 |
| CALCR | 2 | 7 | 18286270 | 18303190 | IGF1 | 5 | 12 | 26272042 | 26353449 | RAG1 | 1 | 11 | 35384554 | 35384554 |
| CARD15 | 3 | 16 | 4348058 | 4373746 | IGF1R | 6 | 15 | 762705 | 953687 | RB1CC1 | 3 | 8 | 5408421 | 5480755 |
| CASP10 | 1 | 2 | 52260093 | 52260093 | IGF2 | 3 | 11 | 944171 | 945420 | RERG | 10 | 12 | 8019967 | 8135603 |
| CASP3 | 4 | 4 | 17961070 | 17973117 | IGF2AS | 2 | 11 | 954784 | 956351 | RET | 2 | 10 | 999281 | 1010000 |
| CASP8 | 2 | 2 | 52339724 | 52341003 | IGF2R | 5 | 6 | 2741319 | 2804822 | RGS17 | 2 | 6 | 57520198 | 57594617 |
| CASP9 | 3 | 1 | 2213456 | 2227198 | IGFBP1 | 1 | 7 | 45304115 | 45304115 | RGS5 | 1 | 1 | 13525818 | 13525818 |
| CASR | 6 | 3 | 28418009 | 28498915 | IGFBP2 | 3 | 2 | 67694106 | 67734402 | RGS6 | 3 | 14 | 53412557 | 53425275 |
| CAT | 4 | 11 | 33246759 | 33281042 | IGFBP3 | 1 | 7 | 45328495 | 45328495 | RNASEL | 2 | 1 | 32952270 | 32963496 |
| CAV1 | 7 | 7b | 15588122 | 15627130 | IGFBP5 | 2 | 2 | 67763629 | 67770883 | ROS1 | 4 | 6 | 21808848 | 21906819 |
| CBR1 | 2 | 21 | 23104502 | 23105435 | IGFBP6 | 2 | 12 | 15631499 | 15636693 | RXRB | 2 | 6 | 24020332 | 24024284 |
| CBR3 | 1 | 21 | 23169639 | 23169639 | IL10 | 5 | 1 | 404971 | 412472 | SAT2 | 2 | 17 | 7127251 | 7127620 |
| CBS | 3 | 21 | 1468132 | 1479791 | IL10RA | 2 | 11 | 21432294 | 21434502 | SCARB1 | 4 | 12 | 2700789 | 2749161 |
| CCL5 | 2 | 17 | 8943983 | 8944760 | IL12A | 1 | 3 | 66205256 | 66205256 | SCUBE2 | 3 | 11 | 7838647 | 7874774 |
| CCNA2 | 2 | 4 | 47234252 | 47237348 | IL12B | 1 | 5 | 3552508 | 3552508 | SELE | 1 | 1 | 20110000 | 20110000 |
| CCND1 | 3 | 11 | 323109 | 326936 | IL13 | 2 | 5 | 34407422 | 34410977 | SEP15 | 1 | 1 | 41147701 | 41147701 |
| CCND3 | 2 | 6 | 32761257 | 32763424 | IL15 | 4 | 4 | 67135593 | 67149678 | SEPP1 | 2 | 5 | 42773481 | 42773565 |
| CCNH | 2 | 5 | 37289632 | 37291745 | IL15RA | 4 | 10 | 357590 | 368570 | SFTPD | 2 | 10 | 450238 | 454840 |
| CCR2 | 2 | 3 | 46295 | 48119 | IL1A | 2 | 2 | 2244966 | 2245095 | SHBG | 4 | 17 | 7131066 | 7135141 |
| CCR3 | 2 | 3 | 46246704 | 46248476 | IL1B | 3 | 2 | 2295176 | 2302130 | SLAMF1 | 3 | 1 | 11070370 | 11107058 |
| CCR5 | 2 | 3 | 58934 | 59029 | IL1RN | 2 | 2 | 2594950 | 2596643 | SLC19A1 | 1 | 21 | 2252162 | 2252162 |
| CD14 | 1 | 5 | 1175843 | 1175843 | IL2 | 2 | 4 | 47872613 | 47873111 | SLC23A1 | 2 | 5 | 41130515 | 41134539 |
| CD4 | 1 | 12 | 6783008 | 6783008 | IL3 | 1 | 5 | 33811491 | 33811491 | SLC23A2 | 6 | 20 | 4794682 | 4920505 |
| CD80 | 3 | 3 | 25758826 | 25770771 | IL4 | 2 | 5 | 34423657 | 34428976 | SLC2A1 | 1 | 1 | 13378031 | 13378031 |
| CD81 | 1 | 11 | 1191843 | 1191843 | IL4R | 6 | 16 | 18635174 | 18688866 | SLC2A4 | 1 | 17 | 6784471 | 6784471 |
| CD86 | 1 | 3 | 28291914 | 28291914 | IL6 | 1 | 7 | 22162940 | 22162940 | SLC30A1 | 1 | 1 | 5214167 | 5214167 |
| CDC25A | 1 | 3 | 48155257 | 48155257 | IL6R | 1 | 1 | 4917325 | 4917325 | SLC30A4 | 1 | 15 | 16567901 | 16567901 |
| CDC25B | 1 | 20 | 3727496 | 3727496 | IL7R | 2 | 5 | 35843947 | 35844030 | SLC39A2 | 2 | 14 | 2466621 | 2468991 |
| CDC25C | 1 | 5 | 40082358 | 40082358 | IL8 | 2 | 4 | 3113034 | 3113747 | SLC4A2 | 3 | 7 | 50119491 | 50120912 |
| CDH1 | 1 | 16 | 22423839 | 22423839 | IL8RA | 1 | 2 | 69236918 | 69236918 | SLC6A3 | 3 | 5 | 1401412 | 1437027 |
| CDK4 | 1 | 12 | 20290025 | 20290025 | INSR | 11 | 19 | 7054288 | 7201441 | SOAT2 | 2 | 12 | 15641004 | 15641233 |
| CDK7 | 1 | 5 | 19125611 | 19125611 | IRF1 | 1 | 5 | 34237146 | 34237146 | SOD1 | 1 | 21 | 18701191 | 18701191 |
| CDKN1B | 1 | 12 | 5633891 | 5633891 | IRF3 | 2 | 19 | 22431099 | 22437210 | SOD2 | 1 | 6 | 2402795 | 2402795 |
| CDKN1C | 1 | 11 | 1695640 | 1695640 | IRS1 | 3 | 2 | 77856775 | 77869959 | SOD3 | 1 | 4 | 15477334 | 15477334 |
| CDKN2A | 7 | 9 | 21958159 | 21987872 | JAK3 | 3 | 19 | 9200231 | 9217823 | SRA1 | 3 | 5 | 1094857 | 1099166 |
| CETP | 2 | 16 | 10623587 | 10631861 | JTV1 | 1 | 7b | 5409604 | 5409604 | STAT1 | 1 | 2 | 42051175 | 42051175 |
| CFH | 5 | 1 | 47051172 | 47118713 | KRAS | 6 | 12 | 18117943 | 18156676 | STK11 | 1 | 19 | 1161484 | 1161484 |
| CGA | 2 | 6 | 25615352 | 25618075 | KRT23 | 1 | 17 | 2817164 | 2817164 | SULT1A2 | 1 | 16 | 19916091 | 19916091 |
| CHEK1 | 2 | 11 | 29059882 | 29087611 | LCAT | 2 | 16 | 21588152 | 21591581 | TEP1 | 4 | 14 | 1851869 | 1876093 |
| COL18A1 | 3 | 21 | 2193419 | 2249664 | LDLR | 3 | 19 | 2486983 | 2504846 | TERF1 | 4 | 8 | 25747287 | 25811386 |
| CRP | 2 | 1 | 10172588 | 10173793 | LEPR | 4 | 1 | 19717140 | 19877906 | TERF2 | 2 | 16 | 23011592 | 23016451 |
| CSF1R | 3 | 5 | 10613068 | 10620614 | LIG1 | 4 | 19 | 20890565 | 20937020 | TERT | 5 | 5 | 1243744 | 1307949 |
| CSF2 | 1 | 5 | 33826473 | 33826473 | LIG3 | 1 | 17 | 8068555 | 8068555 | TFF1 | 1 | 21 | 780627 | 780627 |
| CSF3 | 1 | 17 | 1898198 | 1898198 | LIG4 | 1 | 13 | 21951589 | 21951589 | TFF3 | 1 | 21 | 727269 | 727269 |
| CSTF1 | 2 | 20 | 20020946 | 20021067 | LIPC | 8 | 15 | 29514232 | 29651520 | TFRC | 1 | 3 | 420545 | 420545 |
| CTH | 4 | 1 | 24696151 | 24723461 | LITAF | 2 | 16 | 2955321 | 2960571 | TGFB1 | 1 | 19 | 14127094 | 14127094 |
| CTLA4 | 5 | 2 | 54940421 | 54948335 | LMO2 | 3 | 11 | 32668137 | 32693725 | TGFBR1 | 2 | 9 | 9218937 | 9231818 |
| CTNNB1 | 3 | 3 | 41181997 | 41219444 | LMOD1 | 1 | 1 | 52278196 | 52278196 | TGM1 | 2 | 14 | 5718355 | 5728134 |
| CTSB | 1 | 8 | 4177472 | 4177472 | LPL | 5 | 8 | 7657740 | 7668891 | TLR2 | 3 | 4 | 79102257 | 79120540 |
| CTSH | 1 | 15 | 50004535 | 50004535 | LRP5 | 3 | 11 | 13388198 | 13502756 | TNF | 4 | 6 | 22400559 | 22402009 |
| CX3CR1 | 2 | 3 | 39247166 | 39247260 | LRP6 | 2 | 12 | 5050453 | 5111040 | TNFRSF10A | 2 | 8 | 1431494 | 1433637 |
| CYP17A1 | 5 | 10 | 23329814 | 23345678 | LTA | 2 | 6 | 22398393 | 22398564 | TNFRSF1A | 1 | 12 | 6300243 | 6300243 |
| CYP19A1 | 8 | 15 | 22293401 | 22425455 | MASP1 | 10 | 3 | 93434114 | 93499341 | TNIP1 | 1 | 5 | 11572413 | 11572413 |
| CYP1A1 | 5 | 15 | 45791548 | 45823718 | MATR3 | 1 | 5 | 41083199 | 41083199 | TNKS | 12 | 8 | 1885333 | 2098521 |
| CYP1B1 | 5 | 2 | 17113103 | 17123794 | MBD2 | 3 | 18 | 33170346 | 33208228 | TP53 | 3 | 17 | 7168801 | 7177401 |
| CYP24A1 | 3 | 20 | 17829825 | 17844426 | MBD4 | 1 | 3 | 35647243 | 35647243 | TP53I3 | 4 | 2 | 3118179 | 3124061 |
| CYP2C19 | 1 | 10 | 15270891 | 15270891 | MBL2 | 7 | 10 | 3077777 | 3084515 | TP73L | 9 | 3 | 96077399 | 96109794 |
| CYP2E1 | 2 | 10 | 494964 | 506716 | MDM2 | 1 | 12 | 31376521 | 31376521 | TSG101 | 4 | 11 | 17288969 | 17335787 |
| CYP3A4 | 1 | 7b | 24653205 | 24653205 | MEST | 1 | 7 | 29555519 | 29555519 | TYMS | 2 | 18 | 662792 | 663086 |
| CYP3A7 | 1 | 7 | 24565711 | 24565711 | MET | 3 | 7b | 15765817 | 15862321 | TYR | 1 | 11 | 1272668 | 1272668 |
| CYP7B1 | 3 | 8 | 17352839 | 17372445 | MGMT | 2 | 10 | 2740214 | 2798929 | UCP3 | 2 | 11 | 3938291 | 3942914 |
| DHDH | 2 | 19 | 21706623 | 21711123 | MLH1 | 2 | 3 | 36993572 | 37010110 | UGT1A1 | 1 | 2 | 614298 | 614298 |
| DHFR | 2 | 5 | 30536587 | 30536715 | MMP1 | 3 | 11 | 6223290 | 6230438 | VCAM1 | 2 | 1 | 55003218 | 55023220 |
| DIO1 | 2 | 1 | 8178426 | 8194963 | MPDU1 | 1 | 17 | 7088525 | 7088525 | VDR | 2 | 12 | 10382981 | 10387865 |
| DRD1 | 1 | 5 | 19679782 | 19679782 | MPO | 1 | 17 | 15001508 | 15001508 | VEGF | 2 | 6 | 34594746 | 34610786 |
| DRD2 | 2 | 11 | 16858702 | 16908767 | MSH2 | 7 | 2 | 26445831 | 26519917 | VIL2 | 2 | 6 | 1489523 | 1496551 |
| DRD4 | 1 | 11 | 576433 | 576433 | MSH3 | 4 | 5 | 30578158 | 30763295 | WDR79 | 2 | 17 | 7189831 | 7190151 |
| EDN1 | 2 | 6 | 3152516 | 3154513 | MSH6 | 2 | 2 | 26825749 | 26839048 | WRN | 3 | 8 | 1245331 | 1345428 |
| EFNB3 | 2 | 17 | 7211057 | 7211114 | MSR1 | 2 | 8 | 3852196 | 3880321 | XPA | 1 | 9 | 7780783 | 7780783 |
| EGF | 2 | 4 | 35396328 | 35422995 | MTHFD2 | 1 | 2 | 53245129 | 53245129 | XPC | 3 | 3 | 14127450 | 14139889 |
| EGFR | 3 | 7 | 54635882 | 54662188 | MTHFR | 3 | 1 | 6392038 | 6400424 | XRCC3 | 2 | 14 | 85165680 | 85179020 |
| ENG | 1 | 9 | 37900803 | 37900803 | MTR | 2 | 1 | 1806351 | 1806465 | XRCC4 | 4 | 5 | 32967023 | 33243241 |
| ENPP1 | 1 | 6 | 36316537 | 36316537 | MTRR | 5 | 5 | 7879304 | 7890833 | XRCC5 | 4 | 2 | 67186444 | 67279792 |
| EPHX1 | 8 | 1 | 2187838 | 2208423 | MX1 | 6 | 21 | 28466549 | 28486603 | ZFPM1 | 1 | 16 | 90248 | 90248 |
| EPHX2 | 1 | 8 | 5776249 | 5776249 | MYBL2 | 4 | 20 | 7344876 | 7389599 | ZNF230 | 1 | 19 | 16783732 | 16783732 |
| ERBB2 | 1 | 17 | 1590301 | 1590301 | MYC | 1 | 8 | 41968318 | 41968318 | ZNF350 | 1 | 19 | 24736012 | 24736012 |

Table S1- Alias names, number of SNPs, chromosome and beginning and ending contiguous positions for the 960 tagSNPs used in the Bayesian clustering analysis.
