# Supplementary material for: Genetic Population Structure Analysis in New Hampshire Reveals Eastern European Ancestry
Source: PLoS One. 2009 Sep 7;4(9):e6928. doi: 10.1371/journal.pone.0006928 (PMC2734429; doi:10.1371/journal.pone.0006928)
Supplement: Table S2 — (0.04 MB DOC) [file pone.0006928.s002.doc]

| **# Pops** | **α_AVE_** | **F_ST_1** | **F_ST_2** | **F_ST_3** | **F_ST_4** | **F_ST_5** | **F_ST_6** | **F_ST_7** | **F_ST_8** | **F_ST_9** | **F_ST_10** | **Mean** |
| --- | --- | --- | --- | --- | --- | --- | --- | --- | --- | --- | --- | --- |
| 2 | 0.5597 | 0.0027 | 0.0152 |  |  |  |  |  |  |  |  | 0.0090 |
| 3 | 0.8514 | 0.0170 | 0.0242 | 0.0289 |  |  |  |  |  |  |  | 0.0234 |
| 4 | 0.8660 | 0.0355 | 0.0385 | 0.0407 | 0.0432 |  |  |  |  |  |  | 0.0395 |
| 5 | 0.8319 | 0.0448 | 0.0518 | 0.0531 | 0.0536 | 0.0577 |  |  |  |  |  | 0.0522 |
| 6 | 0.7406 | 0.0576 | 0.0597 | 0.0620 | 0.0634 | 0.0657 | 0.0699 |  |  |  |  | 0.0631 |
| 7 | 0.6970 | 0.0698 | 0.0737 | 0.0748 | 0.0764 | 0.0785 | 0.0823 | 0.0882 |  |  |  | 0.0777 |
| 8 | 0.6539 | 0.0813 | 0.0845 | 0.0882 | 0.0905 | 0.0921 | 0.0942 | 0.0980 | 0.1144 |  |  | 0.0929 |
| 9 | 0.6191 | 0.0907 | 0.0997 | 0.1017 | 0.1038 | 0.1071 | 0.1092 | 0.1124 | 0.1201 | 0.1350 |  | 0.1088 |
| 10 | 0.6163 | 0.1075 | 0.1153 | 0.1188 | 0.1207 | 0.1235 | 0.1263 | 0.1292 | 0.1369 | 0.1496 | 0.1584 | 0.1286 |

Table S2- Average statistical output from *structure* for 10 runs of each K. F_ST_ 's were sorted from low to high and averaged.
