# Supplementary material for: Genetic Population Structure Analysis in New Hampshire Reveals Eastern European Ancestry
Source: PLoS One. 2009 Sep 7;4(9):e6928. doi: 10.1371/journal.pone.0006928 (PMC2734429; doi:10.1371/journal.pone.0006928)
Supplement: Table S3 — (0.10 MB DOC) [file pone.0006928.s003.doc]

|  | **K3_1** | **K3_2** | **K3_3** | **K4_1** | **K4_2** | **K4_3** | **K4_4** | **K5_1** | **K5_2** | **K5_3** | **K5_4** | **K5_5** | **K6_1** | **K6_2** | **K6_3** | **K6_4** | **K6_5** | **K6_6** | **K7_1** | **K7_2** | **K7_3** | **K7_4** | **K7_5** | **K7_6** | **K7_7** |
| --- | --- | --- | --- | --- | --- | --- | --- | --- | --- | --- | --- | --- | --- | --- | --- | --- | --- | --- | --- | --- | --- | --- | --- | --- | --- |
| **Am_Indian** | 0.951 | 0.292 | 0.277 | 0.844 | 0.261 | 0.897 | 0.136 | 0.307 | 0.943 | 0.362 | 0.040 | 0.872 | 0.043 | 0.335 | 0.314 | 0.426 | 0.878 | 0.947 | 0.318 | 0.616 | 0.027 | 0.880 | 0.378 | 0.954 | 0.541 |
| **Austria** | 0.990 | 0.337 | 0.059 | 0.412 | 0.136 | 0.992 | 0.391 | 0.198 | 0.409 | 0.183 | 0.586 | 0.992 | 0.253 | 0.212 | 0.560 | 0.208 | 0.991 | 0.408 | 0.432 | 0.295 | 0.252 | 0.986 | 0.525 | 0.377 | 0.194 |
| **Belgium** | 0.513 | 0.071 | 0.977 | 0.196 | 0.973 | 0.620 | 0.133 | 0.341 | 0.195 | 0.963 | 0.634 | 0.635 | 0.452 | 0.450 | 0.716 | 0.958 | 0.620 | 0.198 | 0.078 | 0.366 | 0.475 | 0.612 | 0.834 | 0.267 | 0.957 |
| **Ca_Indian** | 0.971 | 0.880 | 0.005 | 0.654 | 0.008 | 0.931 | 0.795 | 0.896 | 0.782 | 0.016 | 0.035 | 0.926 | 0.532 | 0.622 | 0.139 | 0.021 | 0.910 | 0.744 | 0.011 | 0.930 | 0.433 | 0.944 | 0.145 | 0.811 | 0.046 |
| **Canada** | 0.295 | 0.214 | 0.889 | 0.298 | 0.875 | 0.247 | 0.314 | 0.736 | 0.584 | 0.861 | 0.025 | 0.291 | 0.116 | 0.799 | 0.127 | 0.773 | 0.286 | 0.567 | 0.800 | 0.528 | 0.083 | 0.289 | 0.166 | 0.561 | 0.741 |
| **Czech** | 0.743 | 0.280 | 0.452 | 0.491 | 0.531 | 0.735 | 0.299 | 0.496 | 0.503 | 0.606 | 0.145 | 0.785 | 0.976 | 0.343 | 0.029 | 0.691 | 0.767 | 0.275 | 0.137 | 0.598 | 0.975 | 0.792 | 0.029 | 0.345 | 0.706 |
| **England** | 0.619 | 0.195 | 0.740 | 0.925 | 0.486 | 0.523 | 0.123 | 0.011 | 0.881 | 0.518 | 0.741 | 0.526 | 0.276 | 0.024 | 0.497 | 0.643 | 0.578 | 0.923 | 0.675 | 0.030 | 0.327 | 0.537 | 0.620 | 0.902 | 0.676 |
| **Finland** | 0.005 | 0.967 | 0.979 | 0.786 | 0.912 | 0.008 | 0.932 | 0.665 | 0.538 | 0.863 | 0.992 | 0.006 | 0.944 | 0.538 | 0.978 | 0.894 | 0.006 | 0.516 | 0.587 | 0.725 | 0.942 | 0.007 | 0.963 | 0.628 | 0.869 |
| **Fr_Canadian** | 0.878 | 0.178 | 0.347 | 0.674 | 0.244 | 0.817 | 0.120 | 0.277 | 0.768 | 0.270 | 0.408 | 0.832 | 0.345 | 0.341 | 0.736 | 0.245 | 0.849 | 0.705 | 0.426 | 0.443 | 0.265 | 0.896 | 0.874 | 0.694 | 0.231 |
| **France** | 0.663 | 0.773 | 0.090 | 0.727 | 0.053 | 0.571 | 0.670 | 0.767 | 0.644 | 0.035 | 0.676 | 0.494 | 0.215 | 0.942 | 0.902 | 0.020 | 0.522 | 0.757 | 0.921 | 0.750 | 0.211 | 0.433 | 0.831 | 0.606 | 0.012 |
| **Germanic** | 0.438 | 0.924 | 0.091 | 0.742 | 0.089 | 0.399 | 0.888 | 0.778 | 0.627 | 0.075 | 0.888 | 0.396 | 0.939 | 0.504 | 0.744 | 0.080 | 0.391 | 0.561 | 0.222 | 0.565 | 0.912 | 0.441 | 0.699 | 0.610 | 0.088 |
| **Germany** | 0.841 | 0.798 | 0.026 | 0.728 | 0.069 | 0.847 | 0.663 | 0.621 | 0.440 | 0.094 | 0.746 | 0.827 | 0.981 | 0.397 | 0.244 | 0.093 | 0.821 | 0.381 | 0.392 | 0.383 | 0.983 | 0.813 | 0.253 | 0.319 | 0.150 |
| **Greece** | 0.098 | 0.303 | 0.933 | 0.745 | 0.851 | 0.092 | 0.199 | 0.230 | 0.674 | 0.862 | 0.664 | 0.082 | 0.689 | 0.220 | 0.429 | 0.831 | 0.055 | 0.568 | 0.707 | 0.151 | 0.721 | 0.053 | 0.531 | 0.488 | 0.783 |
| **Ireland** | 0.050 | 0.835 | 0.732 | 0.713 | 0.693 | 0.087 | 0.825 | 0.497 | 0.539 | 0.656 | 0.944 | 0.086 | 0.861 | 0.377 | 0.839 | 0.635 | 0.098 | 0.455 | 0.807 | 0.164 | 0.858 | 0.109 | 0.845 | 0.359 | 0.626 |
| **Italy** | 0.847 | 0.022 | 0.957 | 0.274 | 0.967 | 0.856 | 0.017 | 0.010 | 0.352 | 0.963 | 0.536 | 0.816 | 0.412 | 0.030 | 0.598 | 0.975 | 0.831 | 0.274 | 0.407 | 0.005 | 0.506 | 0.822 | 0.654 | 0.299 | 0.974 |
| **Jewish** | 0.929 | 0.580 | 0.121 | 0.529 | 0.049 | 0.931 | 0.571 | 0.665 | 0.368 | 0.037 | 0.650 | 0.934 | 0.761 | 0.502 | 0.664 | 0.023 | 0.908 | 0.327 | 0.931 | 0.578 | 0.782 | 0.867 | 0.638 | 0.191 | 0.023 |
| **Lithuania** | 0.649 | 0.604 | 0.134 | 0.073 | 0.201 | 0.772 | 0.705 | 0.556 | 0.037 | 0.207 | 0.956 | 0.780 | 0.783 | 0.452 | 0.911 | 0.214 | 0.759 | 0.017 | 0.691 | 0.442 | 0.842 | 0.775 | 0.930 | 0.024 | 0.227 |
| **Maritime** | 0.864 | 0.181 | 0.382 | 0.559 | 0.252 | 0.847 | 0.242 | 0.371 | 0.548 | 0.230 | 0.257 | 0.833 | 0.214 | 0.438 | 0.369 | 0.225 | 0.851 | 0.536 | 0.557 | 0.377 | 0.228 | 0.845 | 0.322 | 0.419 | 0.186 |
| **Netherlands** | 0.838 | 0.108 | 0.563 | 0.813 | 0.428 | 0.765 | 0.024 | 0.004 | 0.688 | 0.472 | 0.841 | 0.577 | 0.453 | 0.002 | 0.487 | 0.692 | 0.488 | 0.702 | 0.158 | 0.007 | 0.614 | 0.493 | 0.756 | 0.722 | 0.754 |
| **Poland** | 0.503 | 0.892 | 0.110 | 0.015 | 0.530 | 0.742 | 0.937 | 0.680 | 0.001 | 0.524 | 0.949 | 0.778 | 0.990 | 0.296 | 0.691 | 0.616 | 0.745 | 0.001 | 0.119 | 0.695 | 0.991 | 0.751 | 0.698 | 0.001 | 0.741 |
| **Russia** | 0.480 | 0.986 | 0.019 | 0.001 | 0.324 | 0.639 | 0.999 | 1.000 | 0.000 | 0.345 | 0.388 | 0.762 | 0.891 | 1.000 | 0.266 | 0.280 | 0.704 | 0.001 | 0.702 | 1.000 | 0.820 | 0.602 | 0.160 | 0.001 | 0.338 |
| **Scotland** | 0.654 | 0.805 | 0.180 | 0.585 | 0.137 | 0.618 | 0.807 | 0.823 | 0.529 | 0.165 | 0.153 | 0.616 | 0.589 | 0.777 | 0.097 | 0.209 | 0.677 | 0.542 | 0.530 | 0.758 | 0.550 | 0.684 | 0.077 | 0.480 | 0.266 |
| **Sweden** | 0.335 | 0.296 | 0.746 | 0.821 | 0.703 | 0.291 | 0.149 | 0.244 | 0.947 | 0.820 | 0.088 | 0.386 | 0.746 | 0.261 | 0.091 | 0.736 | 0.352 | 0.899 | 0.566 | 0.131 | 0.688 | 0.338 | 0.068 | 0.894 | 0.704 |
| **Switzerland** | 0.075 | 0.273 | 0.886 | 0.826 | 0.805 | 0.038 | 0.140 | 0.178 | 0.938 | 0.829 | 0.092 | 0.043 | 0.104 | 0.364 | 0.412 | 0.901 | 0.070 | 0.982 | 0.738 | 0.297 | 0.075 | 0.060 | 0.368 | 0.973 | 0.881 |
| **UK** | 0.813 | 0.027 | 0.774 | 0.035 | 0.888 | 0.890 | 0.187 | 0.247 | 0.081 | 0.894 | 0.323 | 0.859 | 0.052 | 0.562 | 0.609 | 0.900 | 0.879 | 0.116 | 0.666 | 0.625 | 0.038 | 0.876 | 0.629 | 0.142 | 0.869 |
| **US** | 0.258 | 0.247 | 0.934 | 0.666 | 0.932 | 0.216 | 0.388 | 0.527 | 0.664 | 0.933 | 0.198 | 0.226 | 0.585 | 0.653 | 0.068 | 0.913 | 0.236 | 0.731 | 0.483 | 0.617 | 0.524 | 0.236 | 0.126 | 0.704 | 0.904 |
| **Wales** | 0.477 | 0.390 | 0.321 | 0.882 | 0.170 | 0.401 | 0.182 | 0.275 | 0.850 | 0.239 | 0.338 | 0.434 | 0.928 | 0.105 | 0.162 | 0.248 | 0.423 | 0.722 | 0.194 | 0.232 | 0.934 | 0.524 | 0.222 | 0.719 | 0.283 |

Table S3- A Spearman's rank correlation was conducted between the q-values from CLUMPP output for 10 *structure* runs for between 2 and 7 populations assumed (K2 to K7). A complete table of p-values is shown with p values less than 0.05 highlighted.
