# Supplementary material for: Genetic Population Structure Analysis in New Hampshire Reveals Eastern European Ancestry
Source: PLoS One. 2009 Sep 7;4(9):e6928. doi: 10.1371/journal.pone.0006928 (PMC2734429; doi:10.1371/journal.pone.0006928)
Supplement: Table S4 — (0.07 MB DOC) [file pone.0006928.s004.doc]

| **Ancestry** | **Pop1** |  | **Pop2** |  | **Pop3** |  | **Pop4** |  | **Pop5** |  | **Pop6** |  |
| --- | --- | --- | --- | --- | --- | --- | --- | --- | --- | --- | --- | --- |
| Am_Indian (32) | 6,1 | 0.42 | 5,0 | 0.33 | 2,0 | 0.42 | 11,0 | 0.35 | 3,0 | 0.33 | 3,1 | 0.34 |
| Austria (5) | 0,0 | NA | 1,0 | 0.31 | 1,0 | 0.32 | 2,0 | 0.37 | 0,0 | NA | 1,0 | 0.5 |
| Belgium (5) | 1,1 | 0.39 | 1,0 | 0.35 | 0,0 | NA | 0,0 | NA | 1,0 | 0.59 | 1,0 | 0.42 |
| Ca_Indian (14) | 1,1 | 0.48 | 1,0 | 0.24 | 4,0 | 0.29 | 5,0 | 0.37 | 1,0 | 0.39 | 1,0 | 0.42 |
| Canada (113) | 17,4 | 0.38 | 10,2 | 0.3 | 15,5 | 0.36 | 16,4 | 0.37 | 20,4 | 0.38 | 14,2 | 0.41 |
| Czech (5) | 0,0 | NA | 1,0 | 0.31 | 1,1 | 0.41 | 0,1 | 0.3 | 0,0 | NA | 1,0 | 0.5 |
| England (355) | 48,15 | 0.38 | 52,9 | 0.34 | 47,4 | 0.35 | 44,7 | 0.39 | 52,16 | 0.37 | 56,5 | 0.38 |
| Finland (7) | 0,1 | 0.35 | 1,0 | 0.36 | 0,0 | NA | 0,1 | 0.29 | 2,2 | 0.52 | 0,0 | NA |
| Fr_Canadian (54) | 7,6 | 0.4 | 4,1 | 0.33 | 6,2 | 0.38 | 11,0 | 0.4 | 7,1 | 0.4 | 4,5 | 0.33 |
| France (173) | 29,5 | 0.39 | 18,5 | 0.33 | 24,4 | 0.37 | 25,5 | 0.4 | 25,5 | 0.4 | 22,6 | 0.36 |
| Germanic (5) | 0,0 | NA | 2,0 | 0.35 | 1,0 | 0.4 | 0,0 | NA | 0,1 | 0.41 | 1,0 | 0.36 |
| Germany (110) | 11,2 | 0.36 | 12,2 | 0.31 | 20,3 | 0.35 | 14,2 | 0.41 | 23,1 | 0.37 | 18,2 | 0.4 |
| Greece (9) | 0,1 | 0.28 | 0,0 | NA | 0,2 | 0.37 | 0,2 | 0.31 | 1,2 | 0.35 | 0,1 | 0.3 |
| Ireland (218) | 31,3 | 0.38 | 31,1 | 0.34 | 24,1 | 0.36 | 34,4 | 0.38 | 38,5 | 0.38 | 43,3 | 0.39 |
| Italy (41) | 6,4 | 0.41 | 6,4 | 0.34 | 3,0 | 0.41 | 2,2 | 0.37 | 4,3 | 0.43 | 2,5 | 0.33 |
| Jewish (6) | 0,0 | NA | 0,0 | NA | 1,0 | 0.31 | 3,0 | 0.31 | 1,0 | 0.5 | 1,0 | 0.38 |
| Lithuania (12) | 1,1 | 0.32 | 1,0 | 0.41 | 1,0 | 0.31 | 3,0 | 0.3 | 2,0 | 0.34 | 3,0 | 0.42 |
| Maritime (6) | 0,0 | NA | 0,0 | NA | 1,1 | 0.29 | 1,1 | 0.34 | 0,0 | NA | 2,0 | 0.35 |
| Netherlands (25) | 5,0 | 0.34 | 8,0 | 0.35 | 0,1 | 0.47 | 3,0 | 0.36 | 4,1 | 0.33 | 3,0 | 0.35 |
| Poland (44) | 2,1 | 0.32 | 3,1 | 0.36 | 5,1 | 0.32 | 4,3 | 0.38 | 7,1 | 0.42 | 9,7 | 0.39 |
| Russia (13) | 2,0 | 0.32 | 0,0 | NA | 2,0 | 0.42 | 1,0 | 0.26 | 2,1 | 0.41 | 3,2 | 0.45 |
| Scotland (157) | 21,2 | 0.41 | 21,0 | 0.33 | 26,1 | 0.35 | 28,2 | 0.4 | 26,0 | 0.37 | 29,1 | 0.36 |
| Sweden (24) | 2,0 | 0.35 | 4,0 | 0.32 | 9,0 | 0.42 | 2,0 | 0.43 | 2,2 | 0.48 | 2,1 | 0.36 |
| Switzerland (7) | 1,2 | 0.39 | 2,0 | 0.33 | 1,0 | 0.46 | 0,0 | NA | 1,0 | 0.34 | 0,0 | NA |
| UK (11) | 3,1 | 0.39 | 3,0 | 0.3 | 0,0 | NA | 0,0 | NA | 1,0 | 0.31 | 3,0 | 0.42 |
| US (42) | 5,1 | 0.4 | 5,1 | 0.31 | 5,1 | 0.39 | 5,1 | 0.41 | 6,2 | 0.34 | 8,2 | 0.39 |
| Wales (24) | 1,0 | 0.35 | 6,0 | 0.32 | 8,0 | 0.35 | 3,0 | 0.41 | 3,0 | 0.38 | 2,1 | 0.36 |

Table S4- Counts and sample sizes for ancestry analysis. Each ancestry with more than 5 individuals reporting is listed with total number of people reporting that ancestry in parenthesis. Each population lists how many individuals reported being part (before comma) or only (after comma) a certain ancestry that had their maximum q value for that population. The average q values are shown in the adjacent column.
