# Supplementary figures and images for: Genetic Population Structure Analysis in New Hampshire Reveals Eastern European Ancestry
Source: PLoS One. 2009 Sep 7;4(9):e6928. doi: 10.1371/journal.pone.0006928 (PMC2734429; doi:10.1371/journal.pone.0006928)

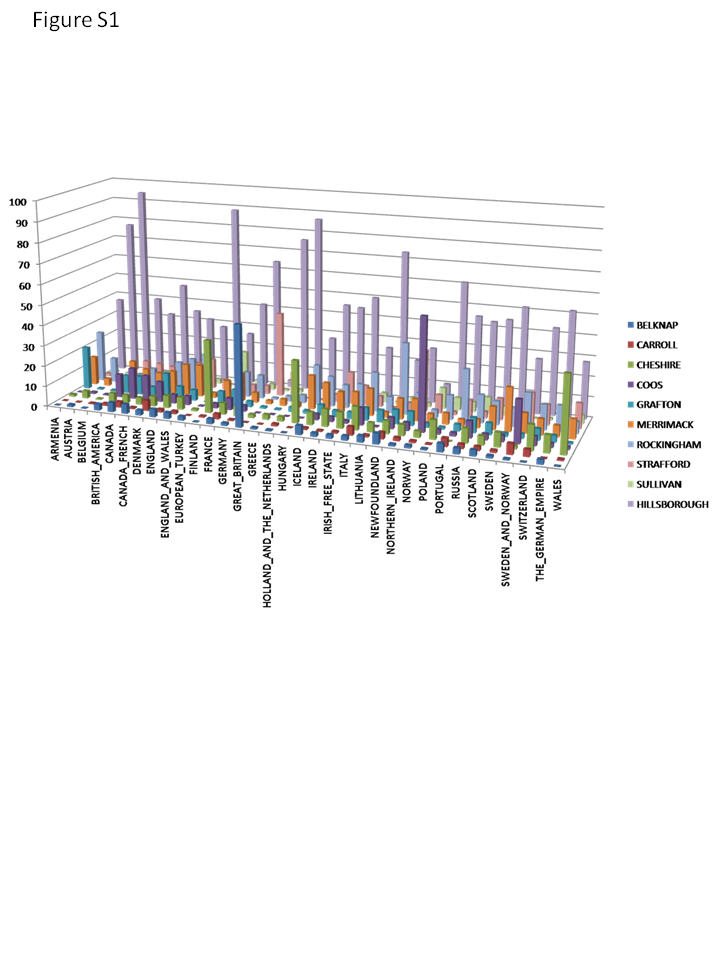

Supplement: Figure S1 — The average number of European immigrants into New Hampshire from 1870 to 1930 reported as percentages of immigrants from each country moving into each county. (0.32 MB DOC) [file pone.0006928.s005.tif]

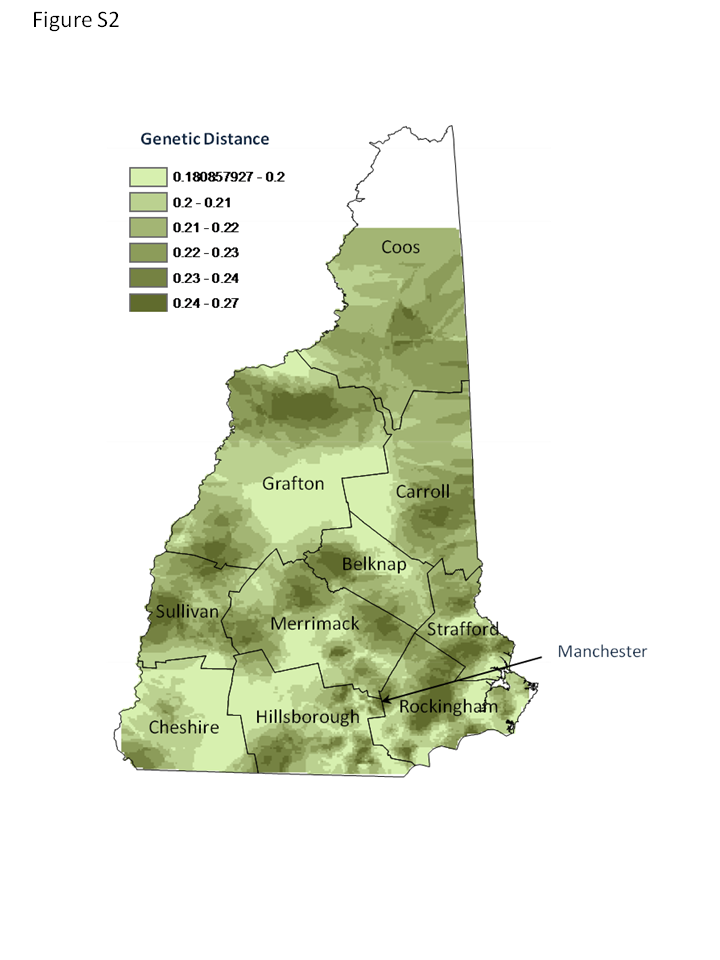

Supplement: Figure S2 — Genetic distance between individuals in New Hampshire using distance values calculated in Alleles in Space, and smoothed using kriging within ArcMap 9.3 (also shows NH county lines). Genetic distances were calculated as the number of mismatched SNPs between individuals connected in a Delaunay triangulation network divided by the total number of SNPs and assigned to the midpoint of the connecting line between individuals. (0.34 MB DOC) [file pone.0006928.s006.tif]
